# Toward an understanding of allogeneic conflict in pregnancy and transplantation

**DOI:** 10.1084/jem.20211493

**Published:** 2022-04-13

**Authors:** Samarth S. Durgam, Maria-Luisa Alegre, Anita S. Chong

**Affiliations:** 1 Section of Transplantation, Department of Surgery, The University of Chicago, Chicago, IL; 2 Section of Rheumatology, Department of Medicine, The University of Chicago, Chicago, IL

## Abstract

Pregnancy is recognized as a spontaneously acquired state of immunological tolerance by the mother to her semi-allogeneic fetus, but it is a major cause of allosensitization in candidates for organ transplantation. This sensitization, assessed by the presence of anti-HLA IgG, contributes to sex disparity in access to transplantation and increases the risk for rejection and graft loss. Understanding this dual tolerance/sensitization conundrum may lead to new strategies for equalizing access to transplantation among sexes and improving transplant outcomes in parous women. Here, we review the clinical evidence that pregnancy results in humoral sensitization and query whether T cell responses are sensitized. Furthermore, we summarize preclinical evidence on the effects of pregnancy on fetus-specific CD4^+^ conventional, regulatory, and CD8^+^ T cells, and humoral responses. We end with a discussion on the impact of the divergent effects that pregnancy has upon alloantigen re-encounter in the context of solid organ transplantation, and how these insights point to a therapeutic roadmap for controlling pregnancy-dependent allosensitization.

## Teaser

Discussion on the divergent immunological effects of pregnancy on T cell and humoral immunity, how prior pregnancy impacts responses upon alloantigen re-encounter in the context of solid organ transplantation, and potential strategies for controlling pregnancy-dependent allosensitization.

## Introduction

The fact that multiple successive pregnancies with the same male partner can be brought to term successfully suggests that the immunological response to a semi-allogeneic fetus is diametrically opposite to the responses elicited by genetically comparable transplanted organs. Peter Medawar in 1953 (Medawar, 1953) discussed this “immunological paradox of pregnancy,” and since then, there have been extensive investigations into how the fetus avoids rejection. A plethora of immune regulatory mechanisms has been uncovered within the uterine environment, including enrichment in regulatory T cells (Tregs), natural killer cells, regulatory macrophages, entrapment of APCs, and chemokine gene silencing of decidual stromal cells (PrabhuDas et al., 2015). Systemic factors that prevent fetal rejection have also been identified, including immune modulation by pregnancy-related hormones and release of tolerogenic placental debris, which may contribute to the preferential systemic expansion of fetus-specific Tregs and acquired dysfunction by conventional T cells (Tconvs) and CD8^+^ T cells. Since the majority of these mechanisms either act locally or only during pregnancy, it was assumed that T cell tolerance would manifest itself only in the context of subsequent pregnancy, and that encounter with the same alloantigens in the context of a solid organ transplant, in the absence of local or systemic pregnancy-induced immunomodulation, would trigger allograft rejection.

The emphasis on T cells as the major mediator of allograft rejection and on T cell tolerance as a means to achieve transplantation tolerance parallels the focus on the constraint of T cells in pregnancy. Thus, despite studies in the 1980s by Bell and Billington (Bell and Billington, 1981; Bell and Billington, 1983; Bell and Billington, 1986) that pregnancy can elicit paternal-reactive antibodies, how pregnancy sensitizes B cell responses while maintaining T cell tolerance to the semi-allogeneic fetus has remained an under-investigated topic in preclinical models (PrabhuDas et al., 2015). In contrast and driven by the ease in quantifying HLA-specific antibodies but difficulty in assessing HLA-specific T cell responses, clinical studies in solid organ transplantation have revealed that pregnancy is a highly sensitizing event that results in the production of fetus-reactive anti-HLA antibodies, and the presence of these antibodies limits access to transplantation and contributes to increased risk of transplant rejection. In this review, we focus on the contrasting effects of pregnancy on these two arms of the adaptive immune system, and on how these pregnancy-shaped responses are recalled by alloantigens that are shared between offspring and transplanted allograft.

## Clinical impact of pregnancy alloimmunization in organ transplantation

### Humoral sensitization

The effect of pregnancy on the immune system was first reported by J.J. Rodd in 1959 when he described peripartum women experiencing an increased number of blood transfusion reactions (Van Rood et al., 1958). It was this observation that allowed for the discovery of anti-HLA antibodies from the sera of pregnant women (Van Rood et al., 1958). Anti-HLA antibodies are produced during the first trimester of a pregnancy and increase in titer over the gestational course and with multiple pregnancies (Lee et al., 2011). During the postpartum phase, antibody levels rise in the first 90 d and gradually disappear in 50% of postpartum women over a 1–2 yr period (Cecka, 2010; Masson et al., 2013). Anti-HLA antibody titers following kidney transplantation increase more robustly in patients having had prior pregnancies than in those having received previous transplantation or transfusion, suggestive of robust pregnancy-induced memory B cells (Higgins et al., 2015). Notably, although pregnancy-induced alloantibodies can diminish with time, alloreactive memory T and B cells can persist (Senn et al., 2021). Thus, anti-HLA antibodies and memory B cells induced by semi-allogeneic pregnancies play a pivotal role prior to and after transplantation, especially for multiparous women.

Historically, anti-HLA antibody titers were measured by the panel-reactive antibody (PRA) technique through a complement-dependent cytotoxicity assay; however, the major limitation of this method is its inconsistency and lack of HLA specificity. In 2009, the United Network for Organ Sharing implemented measuring sensitization using single HLA-coated beads, an assay that precisely identifies specific HLA antigen targets (Cecka, 2010). A computer algorithm generates a calculated PRA (cPRA) according to the HLA frequencies derived from the donor population with the goal of providing consistently accurate results on the extent of sensitization of transplant candidates and the chances for a highly sensitized candidate to find a compatible organ donor. Around 30% of pregnant women are sensitized when measured via complement-dependent cytotoxicity assay, whereas 50–75% of women were found to be sensitized by pregnancy when the single HLA bead assay was used (Bromberger et al., 2017). Furthermore, a retrospective analysis of the United Network for Organ Sharing registry’s waitlist pool showed that individuals with a cPRA >98% were over-represented by women by ~60% (Redfield et al., 2016). Cumulatively, these data reveal the detrimental impact of pregnancy in women in need of a transplant and the disparity it creates toward identifying a suitable donor organ and having a successful post-transplantation course.

Living donor kidney transplantation has better outcomes compared to kidney transplantation from deceased donors (Roodnat et al., 2003). However, 30% fewer women received living donor kidney transplantation as compared with men despite comparable referrals (Bromberger et al., 2017; Roodnat et al., 2003). Pregnancy was identified as a major contributor to this disparity, as postpartum women were increasingly incompatible with their spouse and offspring compared with men (Bromberger et al., 2017). Furthermore, parous women are at a higher risk of being sensitized to unrelated donors sharing an allele of the partner or offspring (Gibney et al., 2006; Vaidya et al., 2006). Child-specific sensitization measured by single-HLA bead assay was detected at the HLA-A/B/C/DR loci in 28–38% of 301 multiparous women analyzed (Honger et al., 2013), with child-specific HLA-B loci being the most sensitizing followed by HLA-A > HLA-DRB1 > HLA-C (Dankers et al., 2003; Honger et al., 2013). Furthermore, by quantifying mother/child mismatches by the number of mismatched HLA eplets, where an eplet is defined as the cluster of amino acids representing the smallest functional unit of structural epitopes on the HLA molecule targeted by B cell receptor and antibodies, the rate of child-specific sensitization increased with the presence of ≥20 mismatched eplets (Honger et al., 2013). These observations are reminiscent of eplet-load mismatch between the organ donor and the recipient predicting de novo anti-HLA antibody production by the host and reduced graft survival, and thus underscories the detrimental effects of pregnancy-induced humoral sensitization (Philogene et al., 2020; Sapir-Pichhadze et al., 2020).

### T cell sensitization

In contrast to the abundant evidence that fetus-specific B cell responses are induced during pregnancy and the barrier they pose to transplantation, the effects of pregnancy-induced effector T cell responses on subsequent transplantation are more opaque. Specifically, although it is clear that maternal T cells acquire tolerance to the semi-allogeneic fetus, it is uncertain whether this T cell tolerance extends to subsequent organ allografts sharing antigens with the fetus. Early observations that fetal-derived stem cells can persist in low numbers in the mother’s circulation for as long as 27 yr, a phenomenon termed peripheral fetal microchimerism (Nelson, 1998), prompted the hypothesis that this microchimerism mediates long-term fetus-specific tolerance in mothers and promotes the acceptance of grafts from their offspring (Starzl et al., 1993). However, several studies testing the correlation between donor/recipient kinship and allograft fate have reported comparable outcomes between groups receiving grafts from offspring versus non-offspring (Cohen et al., 2018; Ghafari, 2008; Mahanty et al., 2001). A recent retrospective analysis performed using the Organ Procurement and Transplant Network living donor liver transplant database revealed that 1-, 5- and 10-yr allografts and patient survival was poorer among mothers who received the organ from their offspring as compared with unrelated living donors (Dagan et al., 2020). A major caveat of such studies is the potential pro-rejection effects of pregnancy-sensitized B cells even when pregnancy-induced antibodies have diminished; as a result, the contribution of pregnancy-primed T cells, either pro-rejection or pro-tolerogenic, may be obscured. Indeed, Senn et al. (2021) reported that women with prior pregnancies receiving kidneys from their husband consistently had a higher rate of antibody-mediated rejection compared with women with prior pregnancies receiving kidneys from other living or deceased donors.

A limited number of studies have attempted to directly quantify ex vivo donor-specific T cell responses arising during normal human pregnancy using proliferation, cytokine production, or cellular cytotoxicity as readouts. When IL-4 and IFNγ ELISPOT assays were used to quantify PBMC responses from non-pregnant versus pregnant women to paternal or pooled alloantigens, Mjosberg et al. (2007) reported that pregnancy did not result in increased paternal-specific IL-4 or IFNγ responses. Furthermore, removal of Tregs resulted in non-specific increases in IFNγ responses and paternal-specific augmentation in IL-4 production. Collectively, their study suggested an absence of pregnancy-specific sensitization of T cells, while also hinting at postpartum Tregs controlling fetus-specific IL-4 responses and broadly controlling IFNγ responses. Notably, reduced frequencies of circulating FoxP3^+^ Tregs were observed with spontaneous preterm birth, preeclampsia, and recurrent spontaneous miscarriages compared to healthy pregnancies suggesting a more systemic effect of Tregs (Dimova et al., 2011; Inada et al., 2015; Inada et al., 2013; Kisielewicz et al., 2010; Koucky et al., 2014; Mjosberg et al., 2010; Nadkarni et al., 2016; Schober et al., 2012; Tilburgs et al., 2008; Tsuda et al., 2018).

Pregnancy-induced Tregs are critical for promoting both primary and secondary pregnancies by suppressing T cell proliferation and cytokine production not only in secondary lymphoid organs but also in the placenta (Salvany-Celades et al., 2019). Expansion of Tregs in the decidual tissue has been prostulated to suppress fetus-specific responses locally (Tilburgs et al., 2008; Erlebacher, 2013). Notably, three different Treg populations have been identified at the maternal–fetal interface: CD25^HI^FOXP3^+^, PD1^HI^FOXP3^−^IL-10^+^, and TIGIT^+^FOXP3^dim^ Tregs. Decidual CD25^HI^FOXP3^+^ Tregs were able to suppress the proliferation and IFNγ and TNFα production by CD4^+^ and effector CD8^+^ T cells in vitro, whereas decidual PD1^HI^ Tregs and TIGIT^+^ Tregs inhibited CD4^+^ but not effector CD8^+^ T cells. However, whether pregnancy-induced Tregs are most potent in the decidua or whether they can also dominantly suppress T cell responses to offspring-matched allografts in secondary lymphoid organs is currently unknown.

CD8^+^ T cell responses to fetus-specific minor antigens have been more consistently reported to develop during pregnancy compared to CD4^+^ T cell responses (Linscheid and Petroff, 2013). Lissauer et al. (2012) assayed fetal-specific CD8^+^ cytotoxic responses using MHC-peptide dextramer multimers bearing a HY-immunodominant peptide in women pregnant with a male fetus. These CD8^+^ T cells expanded during pregnancy and persisted in the post-natal period in 50–62% of pregnant women. Furthermore, the fetal-specific CD8^+^ T cells retained their ability to proliferate, secrete IFNγ, and lyse target cells. These observations corroborated previous studies (Bouma et al., 1996; James et al., 2003; Mommaas et al., 2002; Piper et al., 2007; Verdijk et al., 2004) and suggested that fetal-specific CD8^+^ T cells expand during pregnancy and persist postpartum. It is tempting to speculate that preservation of fetus-CD8^+^ T cell responses during pregnancy, especially in the decidua, may have been evolutionarily selected to ensure the development of protective immunity for the developing fetus against viral infections, given that the fetus is haplo-identical to the mother, and thus maternal HLA-restricted CD8^+^ responses will recognize virally infected fetal cells (Tilburgs and Strominger, 2013; van Egmond et al., 2016). Indeed, observations that the decidua contains a higher percentage of CD8^+^ T cells and a lower percentage of CD4^+^ T cells compared with the peripheral blood is consistent with this possibility (Tilburgs et al., 2009; van Egmond et al., 2016).

Potentially divergent fates of fetus-specific T cell subsets, together with a paucity of studies examining fetus-specific T cell responses in the extended postpartum period, make it difficult to definitively conclude if pregnancy-primed T cells are functionally tolerant or sensitized to fetal antigens presented in the context of a solid organ transplant. The ex vivo quantification of fetus-specific T cell responses is technically challenging and complicated by the increased frequency of pregnancy-induced Tregs (Salvany-Celades et al., 2019). Furthermore, ex vivo observations may not necessarily predict how these cells will behave in vivo after transplantation with organs sharing HLA antigens with the fetus. In vivo studies in postpartum recipients suggest that poorer outcomes are complicated by pregnancy-induced humoral sensitization (Table 1). As a result, proof-of-principle studies conducted in murine models are critical in illuminating the function of fetus-specific T cells upon re-encounter of fetus-matched antigens in the setting of pregnancy followed by solid organ transplantation.

**Table 1.** Retrospective clinical studies assessing the correlation between pregnancy and allograft outcome

Author	No. of transplants	Outcome
Terasaki et al. (1995)	Husband-to-mother: *n* = 368	Comparable allograft survival between spousal donor and unrelated living donor. Pregnancy is a risk factor for loss of allograft
	Child-to-mother: *n* = 1,411	
Mahanty et al. (2001)	Offspring-to-mother: *n* = 874	Fetal tolerance did not translate to a superior allograft survival from offspring donors. Multiple pregnancy trended towards poor allograft survival
	Unrelated living donor to mother: *n* = 310	
Cohen et al. (2003)	Offspring-to-parent: *n* = 3,370	Comparable death censored 5-yr allograft survival in offspring-to-parent compared to unrelated living donor
	Unrelated living donor: *n* = 8,351	
	Deceased donor: *n* = 44,792	
Miles et al. (2008)	Offspring to mother: *n* = 3,124	Comparable and poor allograft survival in offspring-to-parent and parent-to-offspring transplants
	Parent to offspring: *n* = 6,076	
Ghafari (2008)	Offspring-to-mother: *n* = 12	Unrelated living donor allografts survival was significantly higher compared to offspring and husband donor allografts
	Husband-to-mother: *n* = 9	
	Unrelated living donor: *n* = 150	
Choi et al. (2012)	Offspring-to-mother: *n* = 49	Comparable 5- and 10-yr kidney graft survival between offspring-to-mother and offspring-to-father transplant. Mother-to-child had worse outcome
	Parent-to-offspring: *n* = 146	
Redfield et al. (2016)	Highly sensitized: *n* = 7,145	Increased graft loss by 23% among women with a history of pregnancy and transfusion compared to non-sensitized
	Non-sensitized: *n* = 100,147	
Cohen et al. (2018)	Offspring-to-mother: *n* = 1,332	Comparable allograft survival between offspring and unrelated living donor transplant to mother
	Unrelated living donor: *n* = 1,435	
Dagan et al. (2020)	Offspring-to-mother: *n* = 148	Offspring donor allograft survival lower compared to unrelated living donor
	Unrelated living donor: *n* = 93	Male offspring donor resulted in poorer survival compared to female offspring donor
Senn et al. (2021)	Husband-to-mother: *n* = 25	Poor allograft survival among mothers who received allograft from spouse compared to unrelated living donor or deceased donor
	Unrelated living donor: *n* = 52	
	Deceased donor: *n* = 120	

## Semi-allogeneic pregnancy in mice tolerizes T cell responses, but primes fetus-specific B cell responses

### Recognition of fetal antigens by T cells

The identification of fetus-reactive T cells in pregnant mice has relied on either the transfer of a tracer population of TCR-Tg T cells specific for a paternal-derived antigen or the use of fluorescent peptide:MHC multimers. Multimers present a peptide derived from a model antigen such as membrane-bound ovalbumin (mOVA), constitutively expressed by the mating male and present in the seminal fluid and some or all the products of conception (Moldenhauer et al., 2009). During pregnancy, mOVA is expressed in the placental and endovascular trophoblast with access to the decidua and maternal spiral arterioles, respectively, and thus to the gestational mother’s immune system (Erlebacher et al., 2007).

Initial encounter of paternal antigens occurs via exposure to seminal fluid (Moldenhauer et al., 2009), and both CD4^+^ and CD8^+^ T cells reactive to OVA were found in the para-aortic lymph nodes when TCR-Tg T cells were transferred into female hosts immediately postcoitus with an mOVA-expressing male. Using bone marrow chimeric mice, in which only hematopoietic or only non-hematopoietic cells could present OVA, it was shown that presentation of seminal antigen occurred indirectly in female hematopoietic cells (Moldenhauer et al., 2009). During pregnancy, recognition of placental antigen starts at around E10.5, and presentation of paternal antigens by maternal APCs also occurs systemically in secondary lymphoid organs in addition to the para-aortic lymph nodes (Erlebacher et al., 2007). Presentation of fetal antigen increases over the course of gestation, with some presentation remaining until 3 wk postpartum (Erlebacher et al., 2007; Moldenhauer et al., 2009).

### Function of fetus-reactive T cells

While paternal OVA–reactive TCR-Tg CD8^+^ T cells proliferate to cognate antigen expressed in seminal fluid and in placenta, and they persist, they acquire little IFNγ production capacity when compared to positive control stimulation (Erlebacher et al., 2007). Phenotypically, they express high levels of the inhibitory receptor PD-1, and appeared to retain cytotoxic potential but failed to re-expand upon secondary pregnancy (Barton et al., 2017), suggesting a state akin to T cell exhaustion. Indeed, Lewis et al. (2022) used mouse models and human transplant registry data to demonstrate that pregnancy induced a sustained exhausted phenotype in CD8 T cells (PD-1, Lag-3, CD38, Eomes, and TOX) that was associated with hypofunctional CD8^+^ T cells and prolonged allograft survival. A similar profile of high expression of PD-1 and TIM-3 was found in endogenous polyclonal OVA-reactive CD8^+^ T cells of females pregnant with OVA-expressing fetuses (Kinder et al., 2020). This exhaustion state was further reinforced upon secondary pregnancy (but not upon non-pregnancy secondary encounter of the alloantigen), and blockade of PDL1/TIM-3 unleashed the activation of these fetal-reactive T cells and triggered fetal loss during secondary but not primary pregnancy (Kinder et al., 2020).

Analysis of endogenous CD4^+^ T cells reactive to the model antigen 2W expressed by concepti revealed that pregnancy can induce a state of anergy in paternal-reactive Tconvs, characterized by high expression of the surface receptors FR4 and CD73 and lower production of IL-2 upon restimulation (Kalekar et al., 2016). Importantly, a subset of these anergic Tconvs, contained within the Neuropilin-1^+^ population, differentiated into induced Tregs (iTregs) that could suppress inflammation caused by anergic Tconvs reinvigorated during T cell lymphopenia (Kalekar et al., 2016). Both anergic Tconvs and Tregs are thought to be important for the maintenance of fetal tolerance, as depletion of Tregs or blockade of negative regulators of T cell activation expressed by both effector Tconvs and Tregs such as PD-1 are known to precipitate fetal loss (Aluvihare et al., 2004; Guleria et al., 2005); whether these signals are important in Tregs or non-Treg subsets has been difficult to parse out (Zhang and Sun, 2020).

Importantly, pre-existing memory T cells do not cause fetal loss upon antigen-positive pregnancy, despite their relative resistance to Treg suppression (Yang et al., 2007), reduced dependence on co-stimulation for activation (Croft et al., 1994), and ability to enter target tissues in the absence of secondary lymphoid organ priming (Chalasani et al., 2002). It has been shown that OVA-reactive memory T cells generated by immunization with OVA plus adjuvant were prevented from entering the decidua because of epigenetic silencing of CXCL9, CXCL10, and CCL5 in decidual stromal cells, thus suggesting a mechanism of preserving the semi-allogeneic fetus (Nancy et al., 2012).

### Fetus-reactive Tregs

Tregs are necessary for the implantation of early pregnancy following allogeneic mating, but not for sustaining late pregnancy (Shima et al., 2010). Using a pregnancy model in which the concepti express the paternal antigen 2W, Rowe et al. found a preferential expansion of 2W-reactive Tregs over Tconvs, resulting in >60% Tregs of 2W-reactive CD4^+^ T cells by the end of gestation (Rowe et al., 2012b). These Tregs, expanded during primary pregnancy from thymic Tregs and iTregs, persisted after parturition and were recalled rapidly during secondary pregnancy (Rowe et al., 2012b). Indeed, the importance of iTregs in fetal tolerance was confirmed with female mice that lack the FoxP3 CNS1 enhancer element, a necessary region for the differentiation of iTregs. These mice experienced a higher fetal resorption rate when mated with allogeneic compared with syngeneic males (Samstein et al., 2012). In addition, infection with *Listeria monocytogenes* during pregnancy, which elicits inflammation, reduces the ratio of paternal-specific Treg:Tconv, diminishes Treg suppression, and also triggers fetal wastage after allogeneic mating (Rowe et al., 2012a). This could be prevented if the placental entry of effector T cells was blocked with anti-CXCR3 antibody (Chaturvedi et al., 2015). This loss of fetal tolerance during inflammation is similar to the abrogation of transplantation tolerance we observed in recipients of heart allografts infected with *L. monocytogenes* after the establishment of cardiac transplantation tolerance (Wang et al., 2010).

The mechanisms by which antigens in the semen or shed from the placenta can convert Tconvs into iTregs remain to be fully understood. Semen is known to contain high levels of TGFβ (Sharkey et al., 2012), a cytokine which in synergy with IL-2 can drive iTreg differentiation. A recent study suggests that TLR4 signals are essential in the immediate postcoital period to expand Tregs, an observation that may explain why females lacking TLR4 had impaired reproductive outcomes after allogeneic mating (Chan et al., 2021). Interestingly, alloreactive Tregs can potentially arise long before mating, following exposure in utero to maternal tissue that can establish allogeneic microchimerism in the progeny and sustained exposure to non-inherited maternal antigens (NIMA). Subsequent mating of the female offspring with allogeneic males that share determinants with NIMA further expands these Tregs, which confers a more robust fetal tolerance compared with progeny not exposed to NIMA. The increased NIMA-reactive Treg expansion during pregnancy can result in resistance to infection-triggered fetal loss and improved reproductive fitness (Kinder et al., 2015).

The mechanisms by which Tregs prevent fetal wastage are not well understood. Suppression of Tconvs in an antigen-dependent manner likely plays a role, as suggested by the partial loss of 2W^+^ but not 2W^−^ concepti in female mice harboring memory 2W-reactive Th1 cells that are unable to convert into Tregs during subsequent pregnancy (Xin et al., 2014). One mechanism of Treg suppression that may play a role in pregnancy is its ability to induce indolamine 2,3-dioxygenase (IDO) in dendritic cells (Fallarino et al., 2003). IDO causes tryptophan catabolism and kynurenin production that is deleterious to T cell proliferation and survival, respectively. Indeed, pharmacological inhibition of IDO results in fetal loss following allogeneic but not syngeneic mating (Munn et al., 1998). A cautionary observation is that litter sizes of IDO-knockout females mated with allogeneic IDO-knockout males were of normal size, even upon treatment with an IDO inhibitor during gestation (Baban et al., 2004), thus suggesting possible development of compensatory mechanisms when IDO is absent from birth. Finally, Tregs may also prevent fetal loss in a T cell–independent manner, through their control of inflammation, as Treg depletion triggers significant inflammation and fetal wastage, similarly to that observed following injection of LPS (Bizargity et al., 2009). This observation is reminiscent of the loss of cardiac transplantation tolerance in mice with high levels of circulating IFNβ and IL-6 (Wang et al., 2010).

## Semi-allogeneic pregnancy sensitizes fetus-specific B cell responses

Changes in B cell lymphopoiesis occur during pregnancy and have been demonstrated in mice and humans (Lima et al., 2016; Muzzio et al., 2014). Muzzio et al. (2014) reported that immature B cells are lower in number and mature B cells are higher in the bone marrow during the late phase of pregnancy. In the spleen, B220^+^ B cells decreased in number as compared to non-pregnant mice but increased by ∼2–2.3-fold in the para-aortic lymph nodes draining the uterus. Billington and colleagues (Bell and Billington, 1981; Bell and Billington, 1983; Bell and Billington, 1986) demonstrated that murine pregnancy induced anti-paternal alloantibodies in some responder strains of mice, which could be eluted from the placenta and detected in the fetus. Importantly, paternal-specific antibodies increased during the final 3 d of pregnancy and reached maximal levels around 1 wk postpartum, while in secondary pregnancies, the antibody response was observed between day 9 and 10 of pregnancy, consistent with a recall response (Roe and Bell, 1982). While only a limited set of responder mouse strains generated anti-paternal antibodies and only after multiple pregnancies, it is notable that the agglutination or hemadsorption assays used to detect antibodies were relatively insensitive and would only be able to detect high titer antibodies. Additionally, the detection of anti-paternal antibodies was hampered by their lack of complement-dependent cytolytic activity, thus precluding the use of hemolytic assays (Bell and Billington, 1980).

**Figure 1.**
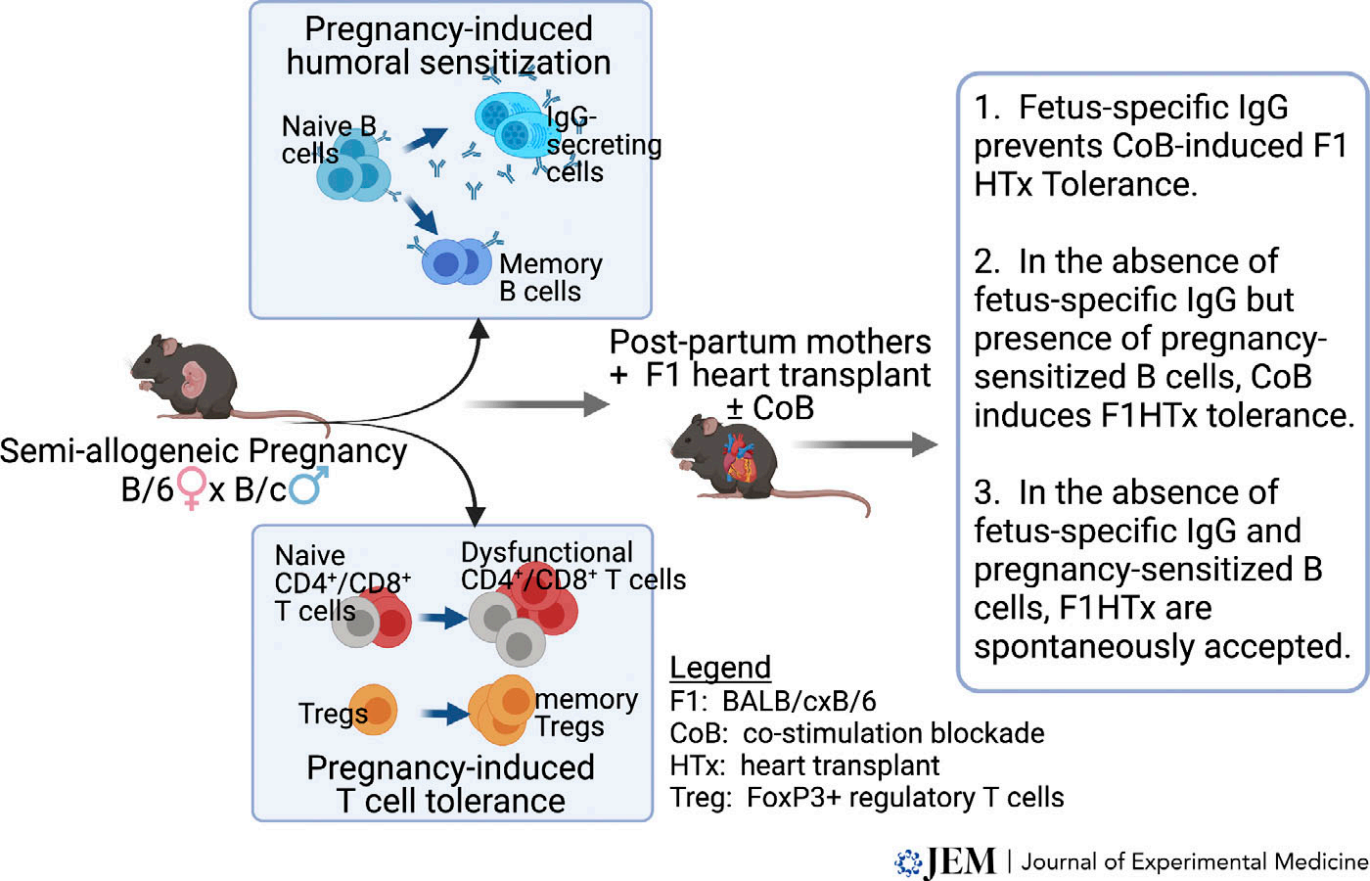
Pictorial summary of the impact of semi-allogeneic pregnancy on fetus-specific T and B cell responses and subsequent consequence upon recall by fetus-matched transplanted allografts.

Recently, Suah et al. (2021) used a mouse model of semi-allogeneic pregnancy that included the 2W1S-OVA as a model paternal antigen to show that 2W-specific T cell responses are tolerized whereas B cell responses are simultaneously elicited during allogeneic pregnancy (Fig. 1). Fetus-specific CD4^+^ Tconvs expanded and developed a phenotype of exhaustion/anergy with upregulated FR4, CD73, and PD-1, and the ability of fetus-specific CD4^+^ and CD8^+^ T cells to produce IFNγ was inhibited as late as postpartum day 45. Importantly, there was a preferential expansion of 2W-specific FoxP3^+^Tregs over Tconvs, and these Tregs exhibited significant increases in the expression of CTLA-4 and CD73. Interestingly, fetus-specific antibody was detected at the time of parturition and increased further in the first week postpartum for most first-time mothers and remained elevated thereafter; all mothers developed fetus-specific antibodies by day 7 postpartum in secondary pregnancies. Thus murine pregnancies recapitulate the humoral sensitizing effects observed in human pregnancies, with the caveat that the fetus-specific antibody response was significantly reduced compared with skin sensitization. Fetus-specific antibodies were generated independently of germinal center reaction but were nevertheless blocked with CTLA-4Ig administered starting at the last week of pregnancy. These observations suggest that the anti-fetus IgG response is T cell but germinal center–independent, raising several questions including the affinity and specific antigenic targets of antibodies, signals provided by T cells to the development of anti-paternal antibodies, and how such helper T cells can develop in the backdrop of T cell tolerance to the semi-allogeneic fetus.

## Pregnancy-induced T cell tolerance to fetus-matched allografts is overridden by sensitized B cell responses

The consequences of pregnancy-sensitized B cell responses to transplanted organs expressing alloantigens shared with offspring is well characterized; however, the implications of pregnancy-induced tolerance of T cell responses to the same allografts are less understood. Pioneering studies by Barton et al. (2017) showed that OVA-specific OT1 T cells adoptively transferred into pregnant mice acquired a state of dysfunction that was persistent when the postpartum mice were challenged with OVA-expressing skin grafts. In addition, those OT1 cells expressed elevated PD-1, and did not expand nor develop the ability to produce cytokines. Nevertheless, postpartum mice were able to reject OVA-skin grafts suggesting that pregnancy-tolerized OT-1 or endogenous OVA-specific T cells retained sufficient function to mediate skin graft rejection. More recently, Kinder et al. (2020) investigated the fate of an endogenous population of fetus (OVA)-specific CD8^+^ T cells in females pregnant after mating with OVA^+^ males. OVA-specific CD8^+^ T cells were primed and accumulated during primary pregnancy and persisted as an activated memory pool after parturition. While the dysfunctional state was reinforced during secondary pregnancies, postpartum mice challenged with OVA^+^ splenocytes in vivo underwent robust expansion and exhibited cytolytic activity. Moreover, in the setting of tumor immunity, Jasti et al. (2017) reported enhanced immune response in post-parous mice bred with OVA-expressing males to subsequent tumors expressing OVA. That endogenous CD8^+^ T cells primed by pregnancy retained the ability to respond to the same antigens in a non-pregnancy context suggested that CD8^+^ T cell responses in secondary pregnancies are curtailed locally and, despite features of dysfunction, may not be sufficient to facilitate the spontaneous acceptance of offspring-matched transplanted grafts.

**Figure 2.**
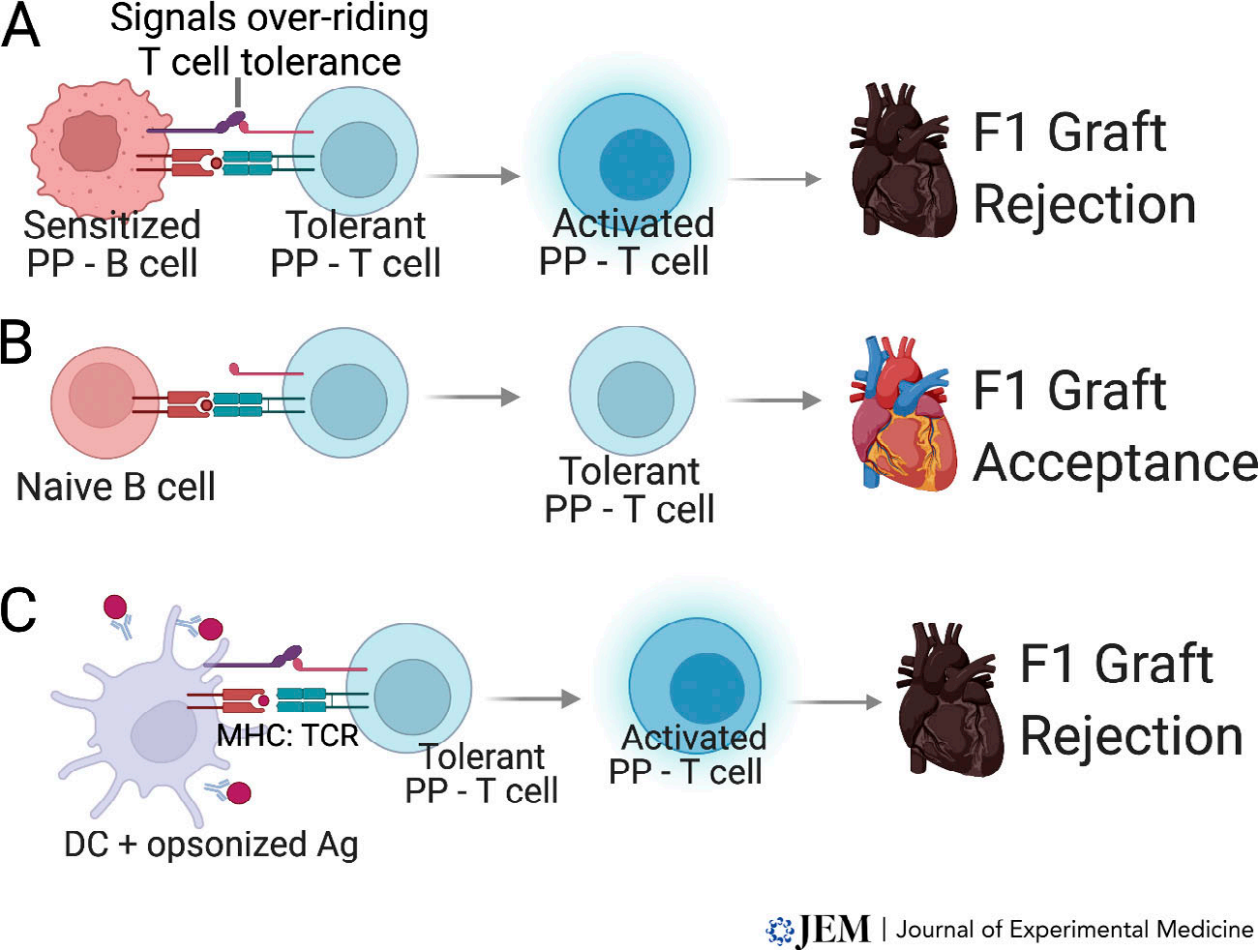
**Postpartum T cell tolerance is overridden in the presence of pregnancy-sensitized B cells and fetus-specific antibodies. (A–C)** Loss of pregnancy-induced T cell tolerance and F1 graft rejection is driven by cognate interactions between pregnancy-primed T and (A) pregnancy-primed B cells but not (B) naive B cells, or (C) with antigen-presenting dendritic cells (DC) activated by donor antigen (Ag) opsonized with pregnancy-primed F1-specific antibodies. The signals overriding T cell tolerance requires definition. PP, postpartum.

The observations of effector/pathogenic T cell responses to fetal antigens encountered in a non-pregnancy context can be explained by an alternative hypothesis, namely that pregnancy-induced T cell tolerance is overridden by pregnancy-sensitized memory B cells and/or fetus-specific antibodies. In support of this hypothesis, postpartum WT females acutely rejected offspring-matched F1 hearts whereas postpartum µMT mice that lacked B cells and antibodies spontaneously accepted F1 hearts. Notably, sIgKO mice that are B cell replete but lacked secreted antibodies and underwent a F1 pregnancy, rejected F1 hearts, while the depletion of B cells restored F1 heart acceptance. Adoptive transfer of pregnancy-primed but not naive B cells from sIgKO mice into postpartum µMT mice precipitated F1 heart rejection (Fig. 2); rejection was associated with the accumulation of fetus-specific Tconvs and restored IFNγ-responses in fetus-specific CD8^+^ T cells. Finally, we showed that anti-fetus antibodies transferred into postpartum µMT mice also precipitated F1 heart rejection; based on our previous observations (Burns and Chong, 2011) we speculate that anti-F1 antibodies function to opsonize antigens, activate antigen-presenting dendritic cells through engagement of Fc, and complement receptors that are able to override T cell tolerance (Fig. 2). These observations are supported by Lewis et al. (2022) where, in the absence of B cells, postpartum µMT mice exhibited prolonged allograft survival compared with virgin µMT mice.

The observations that fetus-specific T cell tolerance induced in the presence or absence of B cells during pregnancy extends to F1 heart allografts only in the absence of pregnancy-primed B cells and antibodies raise new questions. The signals delivered by pregnancy-primed B cells or fetus-specific IgG remain undefined (Fig. 2). Additionally, that pregnancy-sensitized B cells and fetus-specific antibodies do not prevent subsequent pregnancies underscores mechanisms within the placental microenvironment that allow T tolerance to F1 fetus to be maintained but are absent in allografts. Finally, these observations raise the intriguing possibility that humoral desensitization to eliminate pregnancy-primed B cells and antibodies at the time of graft transplantation may reveal a propensity of postpartum women to become tolerant to offspring-matched allografts. Humoral desensitization protocols identified in preclinical models and tested in the clinic suggest a roadmap for exploring this possibility (Alishetti et al., 2020; Jain et al., 2020; Jordan et al., 2021; Schinstock et al., 2020).

## Conclusions

Successful pregnancies balance the need to develop tolerance to the semi-allogeneic fetus and the need to preserve the ability to develop protective immunity to infections in the mother and pass this immunity to the fetus and the neonate. We speculate that preserving humoral immunity, especially late in pregnancy and in the postpartum period, permits the inadvertent generation of fetus-specific memory B cells and antibodies. That these B cells and antibodies are pathogenic for a subsequent organ transplant but do not prevent subsequent pregnancies underscores the potent mechanisms at the maternal–fetal interface that mitigate the effects of antibodies and memory B cells. Understanding these mechanisms might provide novel insights into attenuating their effects in organ transplantation. Conversely, observations that pregnancy-induced T cell tolerance extends to fetus-matched allografts suggest that potent immunomodulatory mechanisms in the uterine–fetal interface are not necessary to constrain fetus-specific T cells in subsequent pregnancies and the possibility of leveraging these pregnancy-induced tolerance mechanisms for promoting the acceptance of allogeneic transplants. Finally, the ability of pregnancy-sensitized B cells and alloantibodies to override potential donor-specific T cell tolerance reveals an opportunity to target humoral desensitization and cognate T:B cell interactions to allow for pregnancy-induced T cell tolerance to dominate in the setting of allograft transplantation.
